# Genetic Deletion of the Transcriptional Repressor NFIL3 Enhances Axon Growth *In Vitro* but Not Axonal Repair *In Vivo*

**DOI:** 10.1371/journal.pone.0127163

**Published:** 2015-05-20

**Authors:** Loek R. van der Kallen, Ruben Eggers, Erich M. Ehlert, Joost Verhaagen, August B. Smit, Ronald E. van Kesteren

**Affiliations:** 1 Center for Neurogenomics and Cognitive Research, Neuroscience Campus Amsterdam, VU University, Amsterdam, The Netherlands; 2 Department of Neuroregeneration, Netherlands Institute for Neuroscience, Amsterdam, The Netherlands; Boston Children’s Hospital and Harvard Medical School, UNITED STATES

## Abstract

Axonal regeneration after injury requires the coordinated expression of genes in injured neurons. We previously showed that either reducing expression or blocking function of the transcriptional repressor NFIL3 activates transcription of regeneration-associated genes *Arg1* and *Gap43* and strongly promotes axon outgrowth *in vitro*. Here we tested whether genetic deletion or dominant-negative inhibition of NFIL3 could promote axon regeneration and functional recovery after peripheral nerve lesion *in vivo*. Contrary to our expectations, we observed no changes in the expression of regeneration-associated genes and a significant delay in functional recovery following genetic deletion of *Nfil3*. When NFIL3 function was inhibited specifically in dorsal root ganglia prior to sciatic nerve injury, we observed a decrease in regenerative axon growth into the distal nerve segment rather than an increase. Finally, we show that deletion of *Nfil3* changes sciatic nerve lesion-induced expression in dorsal root ganglia of genes that are not typically involved in regeneration, including several olfactory receptors and developmental transcription factors. Together our findings show that removal of NFIL3 *in vivo* does not recapitulate the regeneration-promoting effects that were previously observed *in vitro*, indicating that *in vivo* transcriptional control of regeneration is probably more complex and more robust against perturbation than *in vitro* data may suggest.

## Introduction

Successful regeneration of injured axons depends on sufficient extrinsic growth permissiveness and the capacity to activate a neuron-intrinsic gene program that supports axon extension [1–3]. The injured adult CNS is rich in inhibitory factors and injured CNS neurons have a low intrinsic capacity to initiate the regrowth of injured axons [4], in contrast to the PNS [5, 6]. Understanding the interaction between extrinsic growth permissiveness and intrinsic growth potential in the CNS and PNS may help to design new regeneration-promoting therapies that can be used to treat spinal cord injuries, stroke or neurodegenerative diseases [7].

Transcription factors have been put forward as interesting targets for promoting neuron-intrinsic growth properties because they are in principle able to alter the expression of multiple regeneration-associated genes simultaneously and in a coordinated fashion [8, 9]. Previous research for instance showed that neuronal expression of a constitutively active form of the transcription factor CREB can overcome inhibitory environmental constraints and promotes regeneration of lesioned dorsal column axons [10]. Recently we showed that either knockdown or functional inactivation of NFIL3, a transcription factor that is structurally related to CREB, significantly increases axon growth of cultured dorsal root ganglion (DRG) neurons *in vitro* [11]. NFIL3 and CREB are both basic leucine zipper (bZIP) transcription factors that share similar DNA binding domains. Using a combination of transcription factor knockdown or overexpression, luciferase reporter assays, gene expression analysis and chromatin immunoprecipitation we were able to show that CREB and NFIL3 form an incoherent transcriptional feed-forward loop (IFFL) that controls the expression of several regeneration-associated genes [11, 12]. IFFLs are a common transcription regulatory motif used to control both the timing and pulse-like expression of target genes [13, 14]. In DRG neurons, CREB activates regeneration-associated genes, whereas NFIL3, which itself is activated by CREB, acts as a feed-forward repressor of these same genes, thus providing precise temporal control over gene expression.

Our findings suggested that removal of NFIL3 might also increase CREB-mediated transcription and regenerative axon growth potential *in vivo*. To test this hypothesis we generated *Nfil3* knockout mice and quantified functional recovery and injury-induced gene expression following a lesion of the sciatic nerve. Contrary to our expectations we observed that deletion of *Nfil3 in vivo* did not alter the expression of regeneration-associated genes and caused a significant delay in functional recovery following sciatic nerve injury. In addition, dominant-negative inactivation of NFIL3 specifically in DRGs impaired regenerative.

## Materials and Methods

### Animals

Adult male Wistar rats or C57BL/6J mice were used in this study. All animals were individually housed at a 12 h light/dark cycle with ad libitum access to food and water. All animal experiments were approved by the animal ethics committees of the VU University Amsterdam and the Royal Academy of Arts and Science. All surgery was performed under Hypnorm/Dormicum anesthesia, and all efforts were made to minimize animal suffering.

### *Nfil3* knockout mice

*Nfil3* ^lox/lox^ mice were generated using homologous recombination in mouse embryonic stem cells and subsequent blastocyst injection of the appropriately targeted ES cells. The mouse chromosome 13 sequence (nucleotides 53,060,000–53,080,000) was retrieved from the Ensembl database and used as reference. The mouse RP23-12B19 BAC DNA was used for generating the homology arms and conditional region for the gene targeting vector, and for generating Southern blotting probes for confirming correct targeting. The 5’ homology arm (~5.5 kb), 3’ homology arm (~3.5 kb), and conditional region (~3.0 kb) were generated by PCR, cloned sequentially in the FtLoxNwCD vector and confirmed by restriction digestion and end-sequencing. The final vector was obtained by standard molecular cloning. In addition to the homology arms, the final vector also contained loxP sequences flanking the conditional KO region (~3.0 kb), a Neo expression cassette flanked by FRT sequences, and a DTA expression cassette. The final vector was confirmed by both restriction endonuclease digestion and by end sequencing. NotI was used to linearize the final vector prior to electroporation, and 30 μg of linearized vector DNA was electroporated into C57BL/6 ES cells and selected with 200 μg/ml G418. We selected 192 ES clones for PCR based screening and multiple potentially targeted ES clones were identified, expanded, and confirmed by Southern blot analysis to be correctly targeted and harboring a single Neo cassette insertion. Blastocyst injections were performed to create male chimeras for breeding with C57BL/6 wildtype females. To generate *Nfil3* null mice, heterozygous *Nfil3* ^lox/+^ mice were crossed with 129Cre mice expressing Cre-recombinase under the control of a human CMV promoter [15]. The 129Cre mice had been backcrossed to C57Bl/6J mice for at least 10 generations, and the colony of heterozygous *Nfil3* ^+/-^ mice was maintained by backcrossing to inbred C57Bl/6J mice (Charles River Laboratories, L’Arbresle, France) for more than 3 generations before experiments were performed. Male *Nfil3* ^-/-^ knockout mice (further referred to as *Nfil3* KO mice) and *Nfil3* ^+/+^ wildtype littermates (further referred to as WT mice) were used in all experiments.

### DRG neuron cultures and quantification of neurite growth

DRGs were dissected from E13-14 wildtype and *Nfil3* KO mouse embryos, transferred to a 3.5 cm dish (Greiner) containing isolation buffer (Hanks buffered saline solution containing 7 mM HEPES; both from Gibco) and kept on ice. After removal of the nerve roots and other associated tissues, DRGs were transferred to a 15 ml tube containing 4.5 ml isolation buffer 0.5 ml 2.5% trypsin (Gibco) was added and DRGs were incubated at 37°C for 15 min. After 15 min 125 **μ**l DNaseI (4 mg/ml; Roche) was added and DRGs were incubated at 37°C for another 15 min. After 15 min 5 ml DMEM containing 10% ES-FBS and 1% penicillin/streptavidin (all from Gibco) was added to inactivate the trypsin. The cell suspension was then centrifuged for 5 min at 1,000 rpm, the medium removed and cells resuspended in 1 ml culture medium [450 ml MEM (Gibco), 4.4 ml 45% D-(+)-glucose (Sigma), 5 ml GlutaMax (Gibco), 50 ml FBS (HyClone), 50 ng/ml nerve growth factor (Sigma; freshly added from 50 **μ**g/ml stock solution)]. Cells were plated in poly-L-lysine (Sigma)-coated 96-well plates at a seeding density of ~15,000 cells/well and incubated at 37°C/ 5% CO_2_. The following day the medium was replaced by Neurobasal medium [480 ml Neurobasal (Gibco), 4.4 ml 45% D-(+)-glucose (Sigma), 5 ml GlutaMax (Gibco), 10 ml B27 supplement (Gibco), 50 ng/ml nerve growth factor (Sigma; freshly added from 50 **μ**g/ml stock solution)]. Neurobasal medium was replaced every 3 days. Cells were fixed after 1, 5 or 8 days in culture, stained with anti-neurofilament (1:2000; Novus Biologicals, Littleton, CO) and neurite lengths were measured using the Simple Neurite Tracer plug-in in ImageJ (v1.48). Per time point per genotype 5 wells were analyzed.

### Expression constructs and virus production

DN-NFIL3 [11] was subcloned into the AAV-IRES-EGFP construct kindly provided by Dr. Dietmar Fischer (University of Ulm, Germany). As a control, an empty AAV-IRES-EGFP construct was used. AAV serotype 5 (AAV5; plasmid generously provided by Dr. Jurgen Kleinschmidt, University of Heidelberg, Germany) particles were produced and purified using an iodixanol cushion as previously described [16–18]. Viral vector stocks were stored at -80°C in Dulbecco’s phosphate buffered saline with MgCl_2_ and CaCl_2_ (Invitrogen) supplemented with 5% sucrose. Virus titers were determined by quantitative PCR and were typically 1-3x10^12^ genomic copies/ml.

### Rat surgical procedures

Adult male Wistar rats (180–220 g) were anesthetized with Hypnorm/Dormicum (0.08/0.02 ml/100 g I.M.). L4 and L5 DRGs were exposed and injected with 1 μl (1-3x10^9^ genomic copies) virus per DRG using a glass capillary pulled to a fine point and attached to a Hamilton syringe, at a speed of 0.2 μl/min [18]. After the injection the muscle layers were sealed with dissolvable sutures and the skin was closed with Michell-clips. Two weeks later the animals received a sciatic nerve crush. Animals were sedated with 1.8% isoflurane in 0.9 L/min medical compressed air. The sciatic nerve was exposed in the left hind leg and crushed by closing locking forceps with ribbed jaws for 30 sec. The crush site was marked by 10/0 ethilon surgical sutures in the epineurium and afterwards the skin was closed with Michell-clips. One week later animals were sedated with 1.8% isoflurane and the sciatic nerve was exposed again. At 1 cm distal to the crush site the sciatic nerve was transsected and the proximal end was submerged for 30 min in a small cup containing the retrograde tracer FastBlue. Also, part of the distal end of the sciatic nerve was removed for immunohistochemical analysis. Afterwards the nerve was washed to remove excess tracer and the muscle and skin were closed as before. One week later animals were anesthetized with Avertin (250 mg/kg I.P.) and perfused with saline followed by 4% paraformaldehyde (PFA) in saline. Injected DRGs were dissected, post-fixed overnight and transferred to a 25% sucrose solution overnight at 4°C. Tissue was embedded in Tissue-Tek, snap frozen in dry ice-cooled isopentane and stored at -80°C.

### DRG immunohistochemistry and analysis

DRGs were sectioned at 20 μm, post-fixed in 4% PFA, blocked in blocking buffer (PBS containing 1% BSA, 5% FCS and 0.3% Triton X-100) and stained with anti-βIII-tubulin (1:500; clone Tuj1; Covance, Berkeley, CA) and anti-GFP (1:4000; Abcam, Cambridge, UK), followed by donkey anti-mouse Alexa594 (1:400), biotinylated goat anti-rabbit (1:300) and streptavidin-Alexa488 (1:400). All sections were captured at fixed exposure settings using an Axioplan 2 fluorescence microscope (Zeiss, Sliedrecht, The Netherlands) and a 10x objective. Images were analyzed using a custom algorithm in Image-Pro Plus software (MediaCybernetics, Rockville, MD) as previously described [18]. The algorithm automatically identifies all DRG nuclei based on the Tuj1 staining, which specifically stains neuronal cytoplasm and leaves the nucleus unstained and visible as a dark round object. GFP and FastBlue intensities were then measured in a 4-pixel ring around the nucleus. Background intensity levels were measured in non-injected DRGs processed in the same way. Neurons showing >2-fold higher intensities than the mean background level of GFP or FastBlue were classified as transduced and traced neurons, respectively.

### Sciatic nerve immunohistochemistry and analysis

Sciatic nerves distal from the site of tracer treatment were removed, fixed in 4% PFA and sectioned at 20 μm. Sections were blocked in blocking buffer (PBS containing 1% BSA, 5% FCS and 0.3% Triton X-100) and stained with anti-neurofilament (2H3; 1:1000; Hybridoma Bank, University of Iowa, IA). Images were captured at fixed exposure settings using an Axioplan 2 fluorescence microscope and a 20x objective. Surface areas of the different nerve fascicles were calculated using Image-Pro Plus software. The area was then automatically segmented, and segments were randomly selected to sample a minimum of 40% of the total area. In each selected segment, all neurofilament positive axons were counted manually using a light cursor. The total number of regenerating axons was then calculated by multiplying the number of counted axons with the total fascicle area divided by the measured area. The observer was blind for the experimental condition both during image acquisition and image analysis. A two-tailed Student’s t-test was performed to test for significance and a *p*-value <0.05 was considered statistically significant.

### Mouse surgical procedures

Adult male mice were anesthetized with and I.P. injection of Hypnorm (0.1 mg/kg fentanyl citrate/3.3 mg/kg fluanisone HCl) and Dormicum (8.3 mg/kg Midazolam). The sciatic nerve was exposed in the left hind leg, transsected, and the nerve endings were subsequently coaptated using two 10/0 ethilon sutures and the skin was closed with Michell-clips. Throughout and after the operations mice were kept warm until they regained consciousness, and Temgesic (0.01 ml/100 g S.C.) was administered 1x per day until 3 days after the operations. After the experiment mice were sacrificed by CO_2_/O_2_ suffocation.

### Microarrays

L4 and L5 DRGs were dissected at day 2 and day 5 after sciatic nerve lesion and immediately frozen on dry ice. Control tissue was taken from the non-injured side. RNA was isolated using TRI Reagent (Molecular Research Center, Inc., Cincinnati, OH) according to the manufacturers guidelines. RNA quality control was performed using an RNA 6000 Nano chip (Agilent, Santa Clara, CA) on the Bioanalyzer (Agilent, Santa Clara, CA), and concentrations were determined with the Nanodrop ND1000 spectrophotometer (Thermo Fischer Scientific, Waltham, MA). RNA samples were amplified, labeled and hybridized to Agilent 4x44K whole mouse genome microarrays using standard Agilent protocols (Agilent). For each condition we used four independent replicates, two labeled with Cy3 and two with Cy5, and single-color intensities were used for further analyses. Arrays were scanned using an Agilent scanner and data were extracted using Agilent Feature Extraction software. Array data were further processed using the R-packages Bioconductor [19] and limma (Linear Models for Microarray Data) [20] for background correction (Edwards) and quantile normalization. For statistical analyses we used the two class unpaired time course approach implemented in SAMR (statistical analysis of microarrays) [21] and created a linear model with the factors time, genotype and time*genotype in limma [20]. Heat maps and hierarchical clusters were generated using gplots [22]. Transcription factor binding site overrepresentation analysis was performed using oPOSSUM 3.0 [23, 24] with the following settings: background genes: all genes present on the microarray, transcription factor profiles used: all vertebrate JASPAR CORE profiles with a minimum specificity of 8 bits, conservation cutoff: 0.4, matrix score threshold: 85%, sequence searched: 2000/0 bp upstream/downstream. Functional annotation clustering was performed using DAVID (Database for Annotation, Visualization and Integrated Discovery) 6.7 [25, 26] using the following settings: Kappa similarity overlap: 3, similarity threshold: 0.50, classification group membership: initial: 3, final: 3, multiple linkage threshold: 0.50. Enrichment thresholds: 1.0. For analysis of differential expression of groups of genes sharing the same ontologies, we first averaged expression values for genes that were measured by multiple probes using reshape2 [27] and plyr [28]. Data was then converted into ‘ExpressionSet’ format using Biobase [19], genes were annotated using the org.Mm.eg.db database [29], grouped into ontologies of similar biological process, molecular function, or cellular component (group-size between 10 and 500 genes), and tested for grouped differential expression using globaltest [30].

### Open field test

Animals were introduced into a corner of the white square open field box (50x50 cm, walls 35 cm high) illuminated with a single white fluorescent light bulb from above (200 lx), and exploration was tracked for 10 min (Viewer 2, GmbH, Bonn, Germany). Total distance moved was calculated using Viewer 2 (Biobserve GmbH, Bonn, Germany).

### Accelerating rotarod test

Motor function was evaluated using an accelerating rotarod (Roto-rod series 8, IITC Life Science, Woodland Hills, CA, USA). On day one, mice received two habituation trials of 120 s (acceleration of from 0 to 20 rpm in 120 s) followed by 3 training trials (acceleration of from 0 to 40 rpm in 180 s). On day 2, mice received 5 additional training trials. The maximum Rotation Per Minute (RPM) reached before falling off was the dependent measure.

### Narrow beam test

The narrow beam setup consisted of two platforms (30x30 cm), one exposed and one sheltered from light and containing bedding material, connected by a narrow beam (26 mm x 1 m) at 60 cm above the floor. Mice were trained 3x once per day to traverse the beam. After reaching the sheltered platform mice were allowed to stay there for 1 min before being placed back into the home cage. During crossing mice were filmed with a video camera and afterwards crossing time, paw slips, and total duration of hanging from the front paws (side-hanging) were scored. The observer was blinded for genotype and post-operation time. An error score was computed as follows: 1 point per paw slip, 5 points for 1–5 sec of side-hanging, 10 points for 5–15 sec of side-hanging, and 15 points for more than 15 sec of side-hanging. These values were chosen such that on average the maximum penalty for side-hanging was equal to the maximum penalty for paw slips. After the lesion, a narrower beam (12.5 mm) was mounted on top of the original beam to increase the difficulty of the task. Crossing performance was tested every 2–3 days for 1 month.

### Statistical analysis

Open field, rotarod and narrow beam data and were analyzed with repeated measures ANOVA We considered *p*-values <0.05 as significant. Data were log-transformed in situations where Mauchly’s sphericity test was not met, and when this was not sufficient we applied Huynh-Feldt correction. All graphs show mean values ± SEM.

## Results

### Generation of *Nfil3* knockout mice

**Fig 1:**
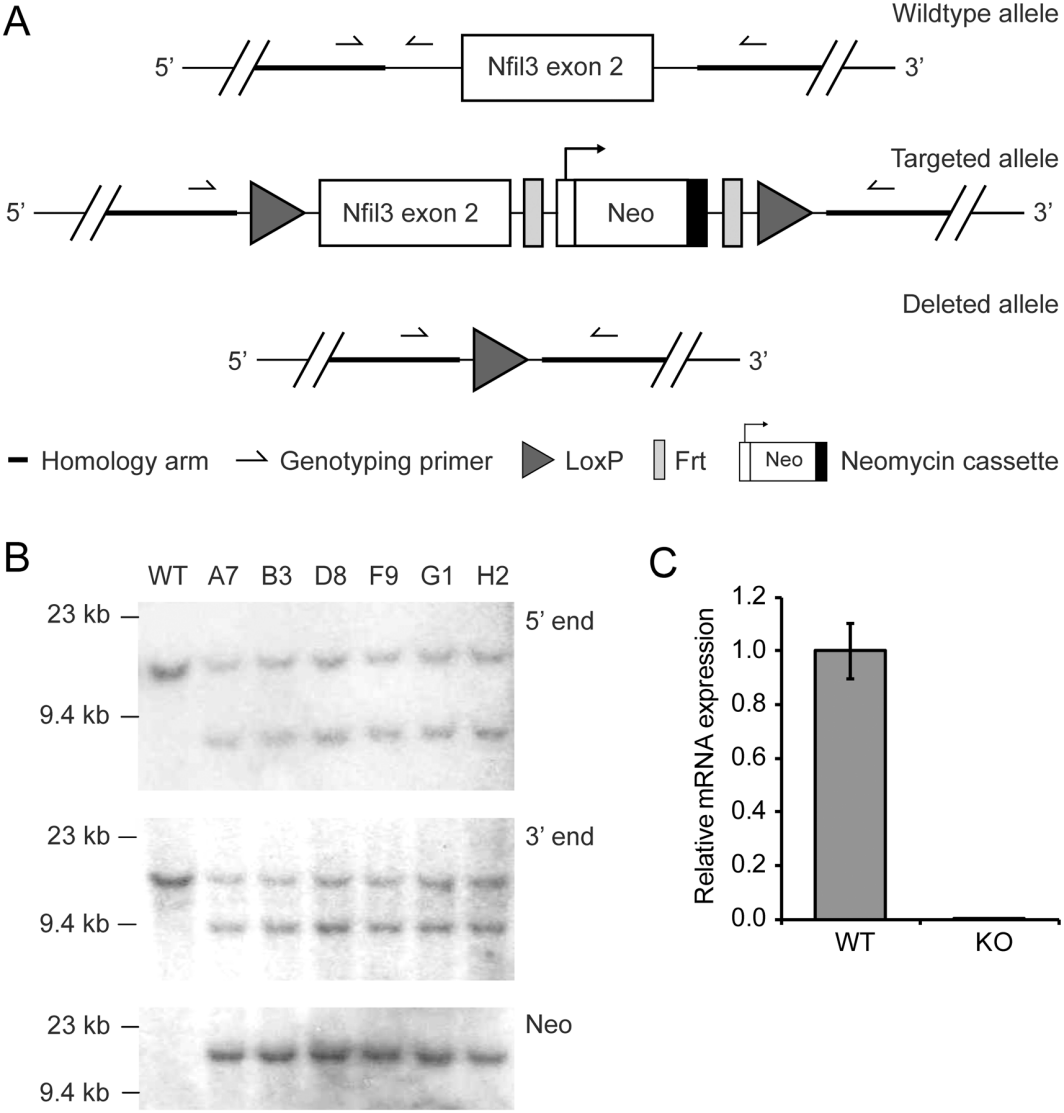
Generation and validation of *Nfil3* KO mice. (a) A schematic representation of the knockout strategy is indicated. (b) Southern blot analysis confirming correct homologous recombination at the 5’ probe side using AvrII digestion yielding fragments of 11.4 kb (wildtype) and 7.6 kb (mutant), at the 3’ probe side using EcoRV digestion yielding fragments of 12.2 kb (wildtype) and 8.9 kb (mutant), and at the Neo cassette using NheI digestion yielding a band of 12 kb (mutant only). (c) *Nfil3* mRNA levels in *Nfil3* KO and wildtype brains as measured by quantitative real-time PCR. Gene expression was normalized against Gapdh expression.

To investigate the role of NFIL3 in axon regeneration we generated conditional *Nfil3* KO mice using homologous recombination in embryonic stem cells (Fig 1a). We confirmed correct insertion of LoxP sites and the Neo cassette by Southern blot analysis (Fig 1b). To generate full KO mice, conditional KO mice were crossed with 129Cre mice expressing Cre-recombinase in germ cells [15]. Absence of Nfil3 mRNA was confirmed by quantitative real-time PCR (Fig 1c).

### DRG neurons lacking *Nfil3* show enhanced axon growth *in vitro*

**Fig 2:**
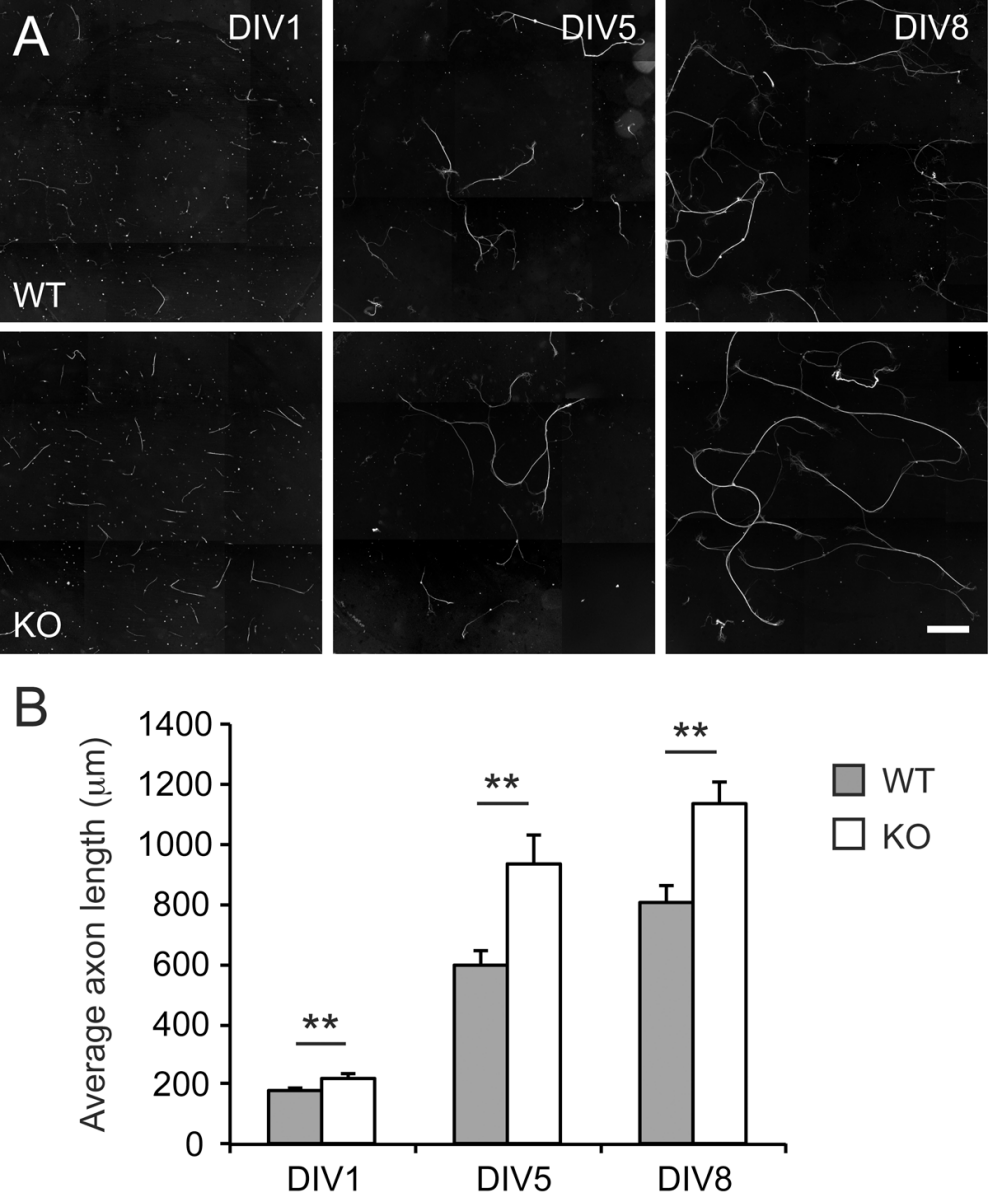
*Nfil3* deletion enhances axon growth of DRG neurons in culture. (a) Example images of cultured embryonic DRG neurons from wildtype mice (top panels) and *Nfil3* KO mice (bottom panels) at 1, 5 and 8 days *in vitro* (DIV; scale bar: 500 **μ**m). (b) Quantification of axon lengths showed that the average axon length of *Nfil3* KO neurons was significantly higher compared to wildtype neurons at DIV1 (222±10 **μ**m vs. 178±9 **μ**m; n = 69/71), at DIV5 (931±101 **μ**m vs. 595±48 **μ**m; n = 26/31) and at DIV8 (1134±77 **μ**m vs. 806±57 **μ**m; n = 32/34) (Student’s *t* test; mean ± SEM; **p < 0.01).

We previously showed that reducing *Nfil3* expression in either DRG neurons or DRG-derived F11 cell using RNA interference significantly increases axon growth [11]. To demonstrate unequivocally that enhanced axon growth is due to lack of *Nfil3* we investigated the growth characteristics of cultured DRG neurons from *Nfil3* KO mice and wildtype littermate controls *in vitro*. Dissociated embryonic DRG neurons were plated in 96-well plates and cultured for 1, 5 or 8 days. Cells were then fixed and stained with anti-neurofilament. Quantification of axon lengths revealed a significant increase in neurite outgrowth in KO cultures compared with cultures prepared from wildtype littermates at all three time points (Fig 2a). Specifically, the average axon length of *Nfil3* KO neurons was 25% higher at DIV1 (222±10 **μ**m vs. 178±9 **μ**m; n = 69/71), 56% higher at DIV5 (931±101 **μ**m vs. 595±48 **μ**m; n = 26/31) and 41% higher at DIV8 (1134±77 **μ**m vs. 806±57 **μ**m; n = 32/34) (Fig 2b). This increase in axon lengths is comparable to what we previously observed when we knocked down the expression of *Nfil3* in either dissociated DRG neurons or in F11 cells [11]. We conclude that genetic deletion of *Nfil3* in DRG neurons enhances axon growth.

### *Nfil3* knockout mice show impaired functional recovery after sciatic nerve lesion

**Fig 3:**
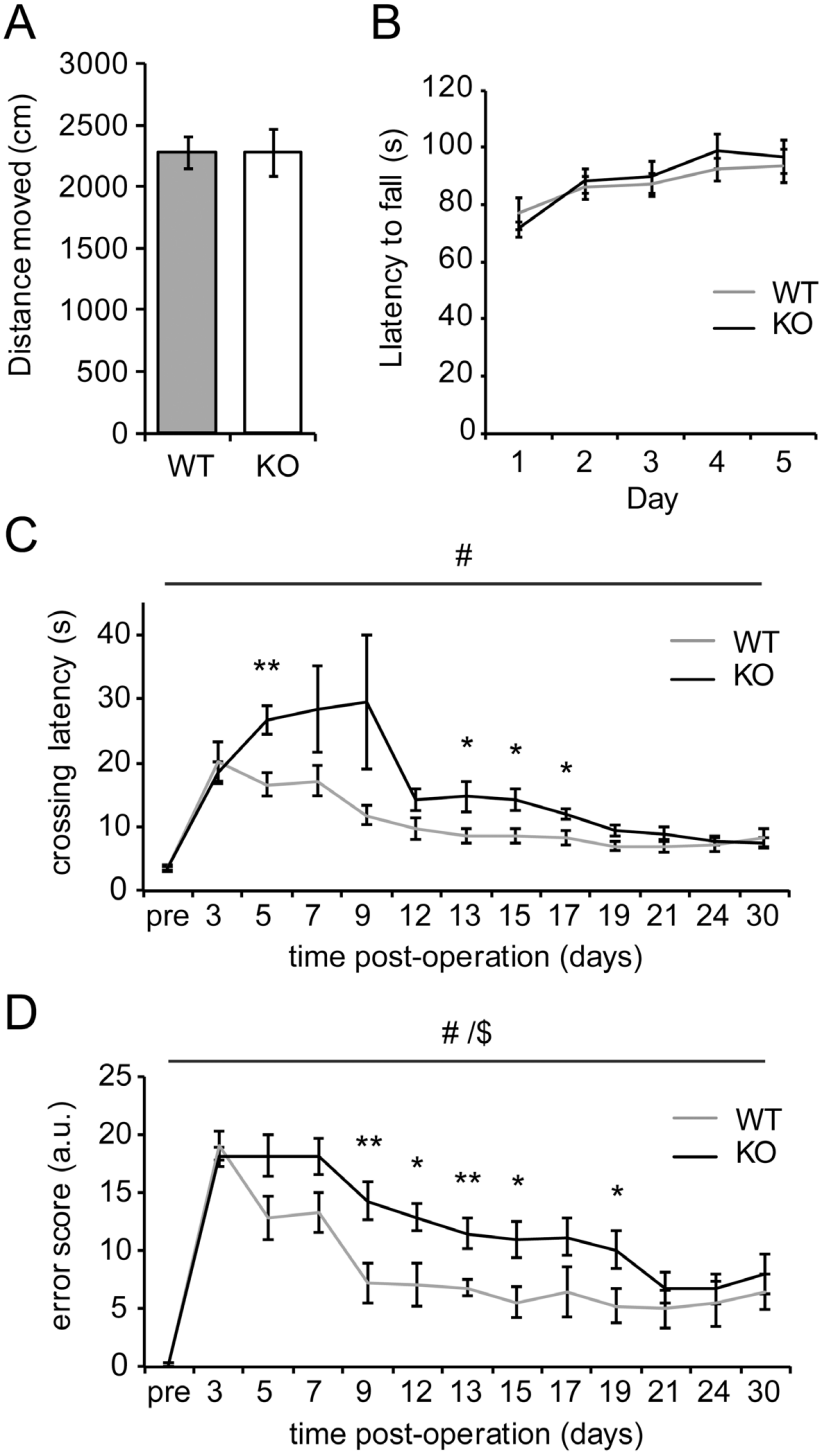
*Nfil3* deletion impairs functional recovery from peripheral nerve injury *in vivo*. (a) *Nfil3* KO mice show no differences in the total distance moved in the open field task (n = 12, *t*_(22)_ = -0.27, *p* = 0.98). (b) The latency to fall off an accelerating rotarod was not affected in *Nfil3* KO mice (n = 12, F_(1,22)_ = 1.02, *p* = 0.32). (c) *Nfil3* KO mice have a significantly longer beam crossing latency than wildtype mice (main effect genotype, n = 11/10, F_(1,19)_ = 8.893, ^#^ *p* = 0.008). Post-hoc t-tests indicated indicate significant differences in performance at post-lesion days 5, 13, 15 and 17 (**p < 0.01, *p < 0.05). (d) *Nfil3* KO mice also make significantly more errors when crossing the beam (main effect genotype, n = 11/10, F_(1,19)_ = 7.145, ^#^ *p* = 0.015; interaction genotype*time, F_(12,228)_ = 2.131, ^$^ *p* = 0.016). Post-hoc t-tests indicate significant differences in performance at post-lesion days 9, 12, 13, 15 and 19 (**p < 0.01, *p < 0.05).

We next wanted to test whether *Nfil3* KO mice also show improved functional recovery from nerve injury *in vivo*. First we measured general locomotor activity in uninjured mice using open field and rotarod tests. The total distance moved in the open field task was unchanged in *Nfil3* KO mice (n = 12, *t*_(22)_ = -0.27, *p* = 0.98, Fig 3a), nor did we observe a difference in rotarod performance (n = 12, F_(1,22)_ = 1.02, *p* = 0.32, Fig 3b), indicating that general movement and motor skills are unaltered in *Nfil3* KO mice. Next, we applied sciatic nerve transsection lesions to *Nfil3* KO mice and wildtype littermates and assessed functional recovery by testing performance in the narrow beam task every other day. As indicators of functional recovery we measured crossing latencies (Fig 3c) and we calculated a weighted error score based on paw slips and time spent side-hanging on the bar (Fig 3d; see materials and methods section for detail). Importantly, we observed no differences in crossing latency and error score during pre-training (Fig 3c and 3d), confirming that basal locomotion and motor skills are unaffected in *Nfil3* KO mice. After the training sessions, beam width was reduced from 26 mm to 12.5 mm to increase the difficulty of the task. Following sciatic nerve injury, *Nfil3* KO mice showed significantly decreased performance and slower recovery compared with wildtype littermates, both in crossing latency (genotype, n = 11/10, F_(1,19)_ = 8.893, *p* = 0.008, Fig 3c) and in error score (genotype, n = 11/10, F_(1,19)_ = 7.145, *p* = 0.015; genotype*time, F_(12,228)_ = 2.131, *p* = 0.016, Fig 3d). These data show that *Nfil3* deletion *in vivo* causes a delayed functional recovery from sciatic nerve lesion, despite the increased axon growth of DRG neurons observed in cultured DRG neurons (Fig 2) [11].

### Inactivation of NFIL3 impairs DRG axon growth following sciatic nerve crush *in vivo*

**Fig 4:**
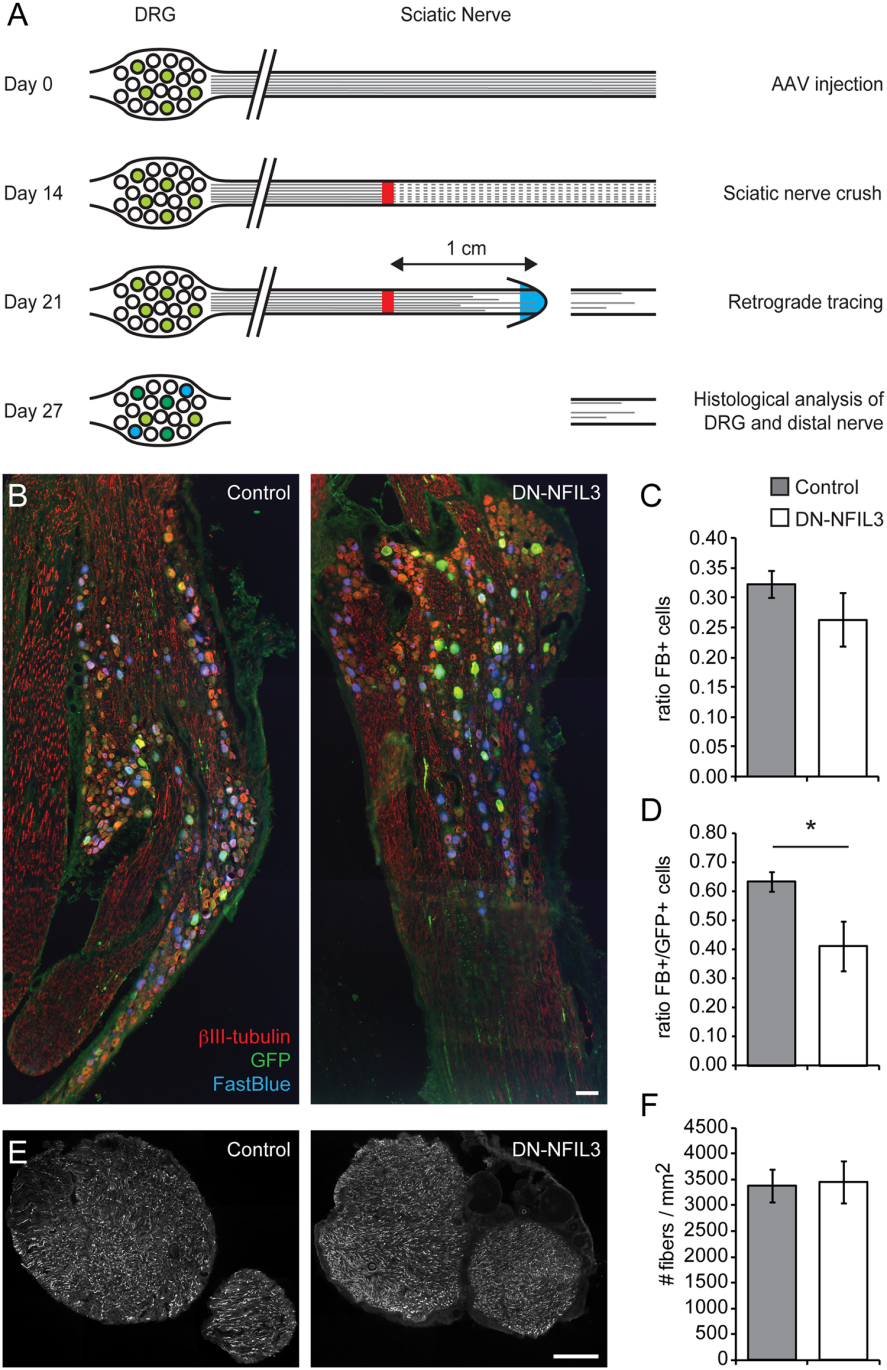
Dominant-negative inhibition of NFIL3 impairs regenerative axon growth *in vivo*. (a) Overview of the experimental design. At day 0 L4/L5 DRGs were injected with AAV5 virus expressing either DN-NFIL3 and GFP, or GFP only. At day 14 the animals received a unilateral crush of the sciatic nerve. At day 21 we transsected the sciatic nerve 1 cm distal from the crush and treated the proximal stump with the retrograde tracer FastBlue. The distal stump was removed for histological analysis. At day 27 animals were sacrificed, and the DRGs were removed for histological analysis. (b) Examples of control and DN-NFIL3 treated DRG sections stained with anti-βIII-tubulin in red, anti-GFP in green, and showing FastBlue labeling in blue (scale bar: 100 **μ**m). (c) The total fraction of FastBlue-positive βIII-tubulin expressing neurons was slightly lower in DN-NFIL3-treated animals compared with controls (n = 8, t_(14)_ = 1.180, *p* = 0.25). (d) When the quantification of FastBlue-positive cells was limited to GFP-positive (i.e. virally transduced) neurons, a significant reduction was observed in DN-NFIL3-treated animals compared with controls (n = 8, t_(9.214)_ = 2.390, *p* = 0.040). (e) Examples of control and DN-NFIL3 treated sciatic nerve sections stained with anti-βIII-tubulin (scale bar: 100 **μ**m). (f) No significant difference was observed in fiber densities in the sciatic nerve at 1 cm distal of the crush (n = 8, t_(14)_ = 0.095, *p* = 0.925).

Because the positive effect of *Nfil3* deletion on DRG axon growth *in vitro* apparently does not translate into improved functional recovery following sciatic nerve crush *in vivo*, we next wanted to know how absence of NFIL3 affects axon regeneration *in vivo*. To allow unequivocal quantification of regenerating fibers in the sciatic nerve, and to exclude possible confounding effects of *Nfil3* deletion in other cell types or systems, we used a retrograde tracing technique combined with dominant-negative inhibition of NFIL3 levels specifically in L4 and L5 DRG neurons in rats. We previously showed that expression of a dominant-negative mutant of NFIL3 (DN-NFIL3) enhances axon growth of rat DRG neurons and DRG-like F11 cells *in vitro* [11]. We injected L4 and L5 DRGs unilaterally with AAV virus expressing DN-NFIL3 and GFP together, or GFP only as control. Two weeks after viral transduction a crush lesion was applied at the level of the sciatic nerve. One week thereafter we transected the sciatic nerve at 1 cm distal of the lesion. The nerve segment distal from this lesion was removed for quantification of regenerating axons, and the proximal nerve stump was loaded with the retrograde tracer FastBlue. One week later, animals were sacrificed and the DRGs were taken out for histological analysis. This experimental procedure is schematized in Fig 4a. DRG sections were stained for βIII-tubulin and for GFP (Fig 4b), allowing quantification of all neurons (βIII-tubulin-positive), transduced neurons (βIII-tubulin- and GFP-positive), and neurons that regenerated an axon beyond 1 cm distal from the crush site (βIII-tubulin- and FastBlue-positive). The total fraction of FastBlue-positive neurons was slightly lower in DN-NFIL3 treated animals compared with controls (Fig 4c), but this difference was not significant (n = 8, t_(14)_ = 1.180, *p* = 0.25). However, when we calculated the number of neurons that was both GFP- and FastBlue-positive, i.e., the fraction of neurons that was actually transduced and had extended axon up until 1 cm distal of the crush site, we found a significant reduction in DN-NFIL3 treated animals compared with controls (n = 8, t_(9.214)_ = 2.390, *p* = 0.040, Fig 4d), indicating that DN-NFIL3 expression reduces axon regeneration *in vivo*. Importantly, no difference was observed in the total number of GFP-positive neurons between treatment conditions (n = 8, t_(14)_ = 1.690, *p* = 0.113). In the sciatic nerve, where fibers from transduced and untransduced cells were indistinguishable, fiber density did not differ between treatments (n = 8, t_(14)_ = 0.095, *p* = 0.925, Fig 4e and 4f). Together these data indicate that, in line with the reduced functional recovery observed in Nfil3 KO mice, regenerative axon growth is impaired in neurons in which NFIL3 function is inhibited. This reduction in regenerative axon growth is specifically observed in neurons that express DN-NFIL3, but the overall effect (i.e., total fraction of FastBlue-positive cells and total number of fibers in the sciatic nerve) is probably masked by the fact that many neurons were not transduced by the virus.

### Established NFIL3 regeneration-associated target genes are not dysregulated in *Nfil3* knockout mice

**Fig 5:**
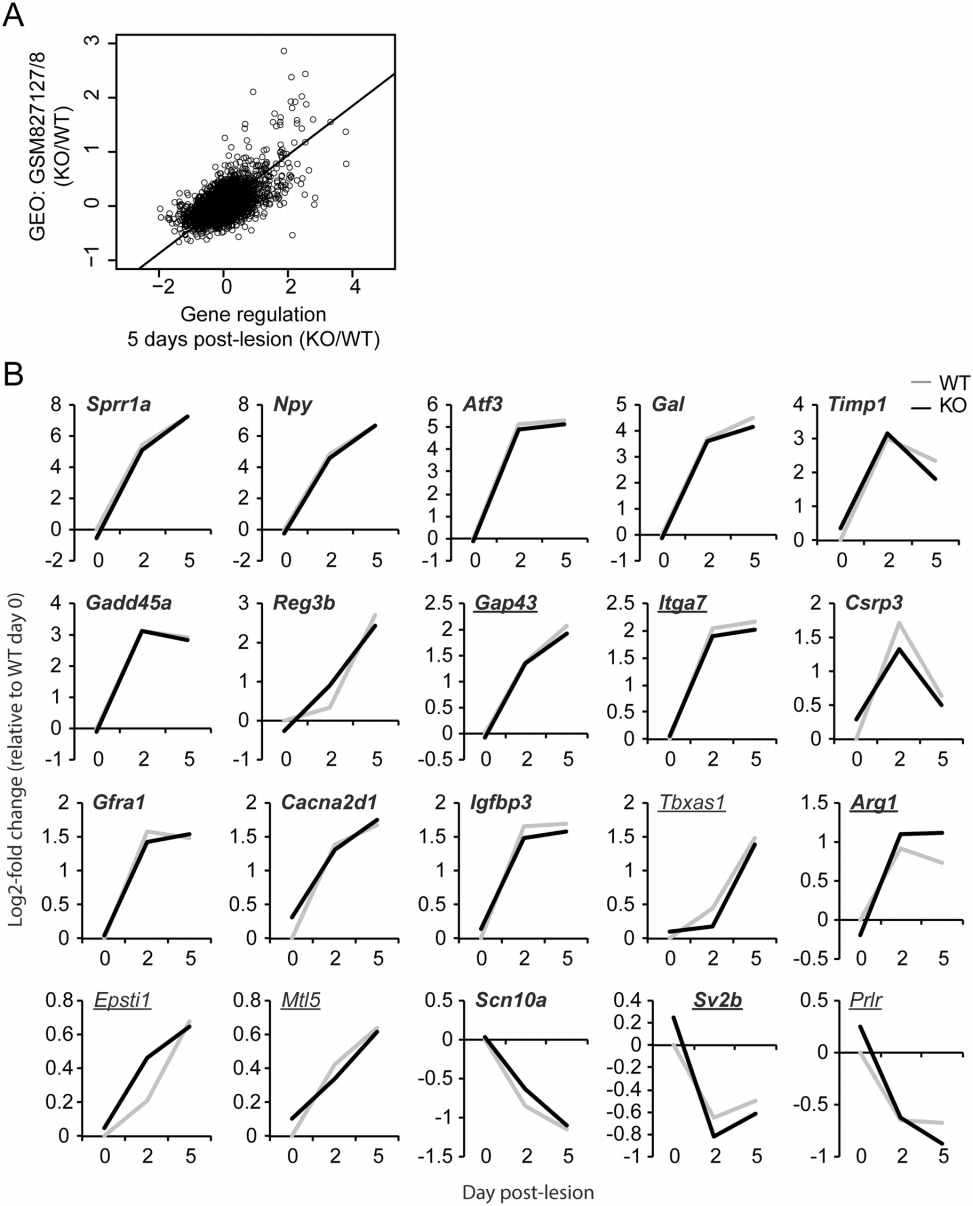
NFIL3 deletion does not alter the expression of regeneration-associated genes. (a) Gene expression was profiled in *Nfil3* KO mice and wildtype controls at 2 days and at 5 days post-lesion, relative to non-injured control DRGs. Gene regulation values in wildtype animals at post-lesion day 5 show a highly significant correlation (r = 0.48, *p* < 2.2x10^-16^) with previously published data [31] describing injury-induced gene expression changes in mouse DRGs at the same time point (GEO datasets GSM827127/8). (b) The expression of well-established regeneration-associated genes and/or NFIL3 target genes is not affected in *Nfil3* KO animals compared with wildtype controls. Of the 20 genes indicated here, 16 are in the core set of regeneration-associated genes identified in three or more independent microarray studies (bold print) [32], and 8 are experimentally validated NFIL3 target genes (underlined) [11, 12]. No significant differences were observed between expression profiles of *Nfil3* KO animals and wildtype controls.

To understand why *Nfil3* deletion does not promote axon regeneration and functional recovery *in vivo*, we next tested the transcriptional role of NFIL3 in injured DRG neurons. We performed mRNA expression microarray analysis on DRGs following sciatic nerve lesion in *Nfil3* KO mice and wildtype controls, using contralateral DRGs as control tissue (n = 4 per genotype per condition; GEO accession number GSE66259). We focused on expression differences that occur relatively early after injury, i.e., at 2 days and 5 days post-lesion, since this is the period when the highest expression levels of *Nfil3* are observed [11]. Using linear modeling we identified 5489 unique genes significantly regulated due to the lesion at either 2 or 5 days post-lesion, independent of genotype (S1 Table). To allow comparison of our findings with previously published regeneration-associated gene expression profiling studies we downloaded data from Kim et al. [31] describing gene expression data in mouse DRGs at 5 days post-lesion compared with uninjured control tissue (GEO sets GSM827127 and GSM827128). We filtered for genes that passed the reported detection test (*p* < 0.05), calculated gene regulation values relative to the uninjured control levels and compared these to our own regulation values in wildtype mice at the same time point (i.e., 5 days post-lesion). We found that the two datasets are significantly correlated (r = 0.48, df = 5236, *p* < 2.2x10^-16^, Fig 5a). These findings indicate that we profiled valid injury-induced and regeneration-associated genes.

We next asked whether *Nfil3* deletion causes a dysregulation of known regeneration-associated genes. We compared expression profiles in knockout and wildtype mice of 20 genes that are consistently found regulated in multiple gene expression studies [32] and/or contain previously identified and experimentally validated NFIL3 binding sites [11, 12]. All these genes showed strong injury-induced regulation over time, but for none we could observe a difference in expression between knockout and wildtype DRGs (Fig 5b). Even *Gap43* and *Arg1*, which we previously showed to bind NFIL3 *in vivo*, show no increased expression in *Nfil3* KO mice compared to WT. From this we conclude that removal of NFIL3 does not de-repress established NFIL3 target genes.

### Genes differentially expressed in *Nfil3* knockout mice

**Fig 6:**
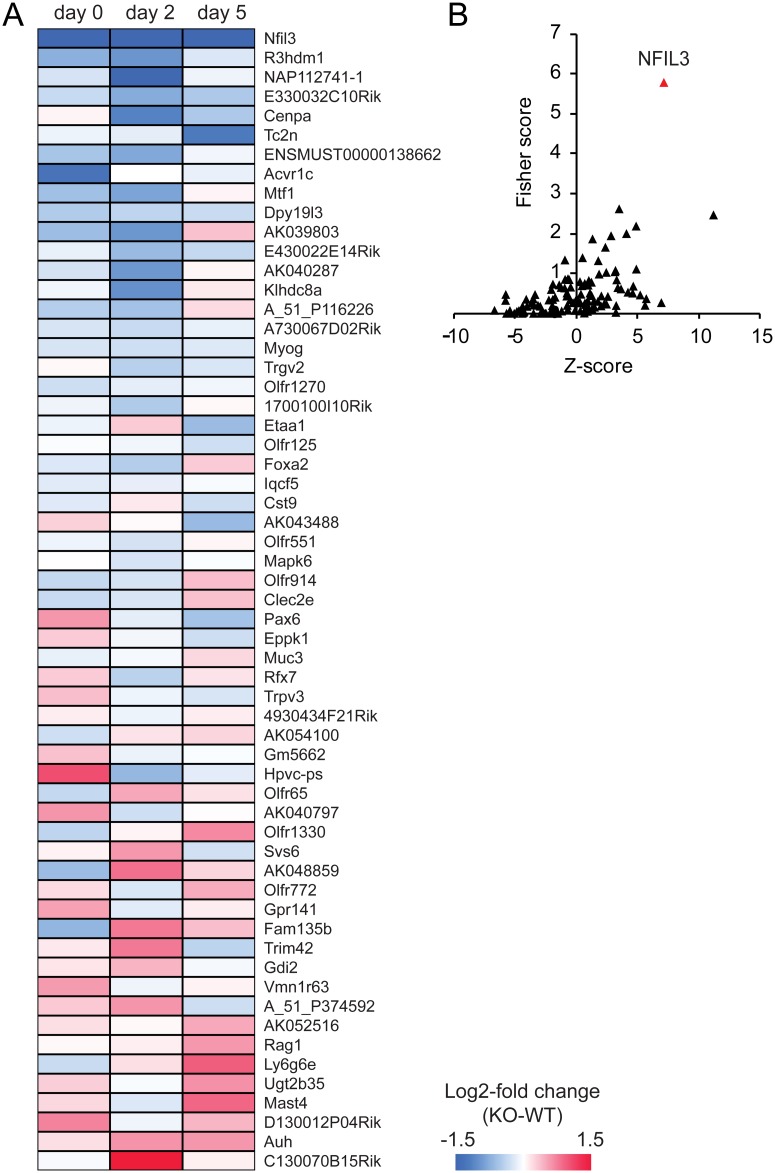
NFIL3 deletion alters the expression of a small set of NFIL3 target genes. (a) The injury-induced regulation of 59 genes was found significantly different in *Nfil3* KO animals compared with controls. Color scale indicates log2-fold expression difference between genotypes. (b) Within the promoter regions of these 59 genes, NFIL3 binding sites are the most overrepresented binding sites judged on the combined Fisher score and Z-score computed by oPOSSUM.

To determine which other genes may be regulated by NFIL3 following sciatic nerve lesion, we used time course analysis in SAM and time*genotype interaction analysis in limma to detect expression profiles that significantly differ between genotypes. We identified 59 genes that were differentially regulated due to the lesion in *Nfil3* KO mice compared with wildtype controls (adjusted *p* < 0.05; Fig 6a). When we tested these genes for overrepresented transcription factor binding sites in 0–2000 bp region upstream of the transcription start site, we found NFIL3 binding sites to be most significantly overrepresented based on the combined Fisher score and Z-score reported in oPOSSUM [23, 24] (Fig 6b), indicating that these genes most likely represent true NFIL3 target genes. Functional enrichment analysis identified two gene ontology terms that are significantly overrepresented within these genes: ‘olfactory signal transduction’ and ‘transcriptional regulation’ (Table 1). Global testing subsequently revealed that at 5 days post-lesion there is a significant dysregulation in *Nfil3* KO mice of multiple genes that are involved in basal transcriptional regulation (Table 2), while no significant overrepresentation of gene ontology terms was detected at day 2 post-lesion. Taken together these data suggest that the *Nfil3* deletion affects the expression of a small set of target genes *in vivo*. These genes include regulators of basal gene transcription, in particular at day 5 post-lesion, as well as several transcriptional regulators that are not known to be involved in axon regeneration.

**Table 1:** Gene ontology terms overrepresented within genes differentially regulated in *Nfil3* KO mice.

Functional class	Gene names	Enrichment score
Olfactory signal transduction	Olfr551, OlfrR65, Olfr1270, Olfr914, Olfr1330, LOC100046375, Olfr64, Gpr141, Olfr125, Vmn1r63, Pax6, Trpv3, Clec2e, Dpy19l3, Uxs1, Acvr1c	1.21
Transcription regulation	Mtf1, Foxa2, Pax6, Myog, LOC100046232, Cenpa, Rfx7, Rag1, Nfil3	1.17

**Table 2:** Gene ontology terms overrepresented within genes differentially regulated at 5 days post-lesion.

GO term	GO description
GO: 0002862	Negative regulation of inflammatory response to antigenic stimulus
GO: 0001012	RNA polymerase II regulatory region DNA binding
GO: 0000977	RNA polymerase II regulatory region sequence-specific binding
GO: 0007623	Circadian rhythm
GO: 0000979	RNA polymerase II core promoter sequence-specific DNA binding
GO: 0001078	RNA polymerase II core promoter proximal region sequence-specific DNA binding transcription factor activity involved in negative regulation of transcription
GO: 0001227	RNA polymerase II core promoter regulatory region sequence-specific DNA binding transcription factor activity involved in negative regulation of transcription
GO: 0001046	Core promoter sequence-specific DNA binding
GO: 0001047	Core promoter binding
GO: 0000982	RNA polymerase II core promoter proximal region sequence-specific DNA binding transcription factor activity

## Discussion

In this study we characterized the role of the transcriptional repressor NFIL3 in axon regeneration following nerve injury using genetic deletion and dominant-negative inhibition. In line with previous findings [11] we show that genetic deletion of *Nfil3* promotes axon growth in cultured DRG neurons *in vitro*. In strong contrast however, genetic deletion or dominant-negative inhibition of NFIL3 *in vivo* did not enhance regenerative axon growth, did not improve functional recovery, and did not alter the expression of known regeneration-associated genes following sciatic nerve lesion. Instead, we observed a reduction in regenerative axon growth, a delay in functional recovery, and a dysregulation of genes not previously linked to axon regeneration.

Many transcription factors are regulated by nerve injury [8]. These transcription factors are interesting targets for promoting regenerative axon growth because they are potentially able to coordinate the expression of multiple regeneration-associated genes simultaneously [9]. Several recent studies indeed showed that inducing the expression or activity of transcription factors provides an efficient way to enhance the regenerative potential of axotomized neurons *in vivo*. Expression of constitutively active CREB, for instance, promotes regeneration of lesioned dorsal column axons [10], and activation of SMAD1, by increasing the levels of BMP2 or -4 *in vivo*, enhances axon growth of adult DRG neurons *in vitro* [33]. Overexpression or activation of a transcription factor is however difficult to control and may result in unwanted side effects, and inactivation of inhibitors of regeneration may provide an even better approach to promote axon regeneration. In this respect, Moore et al. for instance showed that genetic deletion of KLF4, a KLF family transcription factor that inhibits axon growth, enhances regeneration of damaged retinal ganglion cell axons into the optic nerve [34].

We recently showed that NFIL3 is a potent neuron-intrinsic inhibitor of axon growth in injured cultured adult DRG neurons [11]. From a panel of 62 transcription factors that were up- or downregulated during regeneration of DRG neurons after sciatic nerve injury *in vivo*, knockdown of NFIL3 had the strongest outgrowth promoting effect *in vitro*. Moreover, NFIL3 was shown to repress known regeneration-associated genes, including *Gap43* and *Arg1*. Here we could replicate these findings by demonstrating that genetic deletion of *Nfil3* in mice results in a strong increase in neurite outgrowth from cultured DRG neurons compared with wildtype neurons. However, *Nfil3* KO mice did not show enhanced functional recovery from sciatic nerve injury. In fact we observed a significant reduction in crossing latency and error score in the narrow beam task, and our data indicate that maximal recovery in *Nfil3* KO animals is delayed by approximately one week compared with wildtype controls. These data suggest that regenerative axon growth in *Nfil3* KO mice is reduced rather than enhanced. Although basal locomotor activity and motor skills were not affected we cannot exclude the possibility that adverse side effects of the global gene deletion are responsible for the impairment in functional recovery. Specifically, NFIL3 is known to play an important role in immune system development [35–37], and an altered immune response could have contributed to the delay in functional recovery. Therefore we used a DRG neuron-specific dominant-negative approach to test whether inhibition of NFIL3 function affects regenerative growth directly in a cell-autonomous manner. The DN-NFIL3 construct used here was shown to bind to NFIL3 and to increase axon growth in cultured DRG neurons [11]. We transduced neurons in L4 and L5 DRGs and lesioned the sciatic nerve two weeks later. We observed that within the populations of neurons that expressed DN-NFIL3, less neurons were able to extend an axon up to 1 cm beyond the lesion side compared with GFP transduced controls. Due to the relatively low transduction rates (10–15%) this effect was not detected in the total number of traced neurons or in the number of fibers in the distal nerve stump. Taken together however, these observations convincingly show that inhibition of NFIL3 function reduces regenerative axon growth only in neurons that actually express DN-NFIL3.

Given the fact that NFIL3 inactivation reduces axon regeneration *in vivo* in a cell-autonomous manner, whereas previously we showed that either knockdown or dominant-negative inhibition of NFIL3 increases the expression of regeneration-associated genes and enhances axon growth *in vitro*, we next wanted to know how *Nfil3* deletion affects injury-induced gene expression in the DRG. Microarray expression profiling at 2 and 5 days after sciatic nerve lesion revealed the induction of a large set of regeneration-associated genes. However, focusing on the most common regeneration-associated genes, including seven experimentally validated NFIL3 target genes [12], no significant differences were observed in expression profiles between *Nfil3* KO mice and wildtype controls. These findings might explain why we did not find a positive effect of *Nfil3* deletion on axon regeneration and functional recovery, but they do not explain why regeneration in *Nfil3* KO mice is impaired in comparison with wildtype controls, suggesting that two distinct mechanisms are involved. In this respect it was interesting to find that in addition to regeneration-associated genes not being differentially regulated, a small set of other genes did show changes in expression in *Nfil3* KO mice. In particular we found components of olfactory signal transduction pathways and several transcription factors dysregulated. Interestingly, one of the upregulated transcription factors, PAX6, is a regulator of olfactory system development [38], suggesting that the dysregulation of olfactory signaling pathways may be due to a de-repression of *Pax6* in the absence of NFIL3. Induction of this, and possibly also other, gene expression programs that have no role in axon regeneration or may even actively repress axon regeneration might explain the adverse effects of *Nfil3* deletion on regeneration.

There may be several reasons why *Nfil3* deletion promotes axon growth *in vitro*, but not axon repair *in vivo*. Firstly, the timescale on which these processes take place are very different. *In vitro*, adult DRG neurons extend axons over large distances within the first 48 hours in culture, whereas *in vivo* it takes two weeks before functional recovery become apparent. We previously showed that NFIL3 suppresses regenerative axon growth by repressing CREB target genes [11]. CREB activity may thus be necessary to reveal the growth-promoting effects of *Nfil3* deletion, and possibly injured neurons *in vivo* are not able to sufficiently activate CREB target genes long enough to benefit from the absence of feed-forward repression by NFIL3. In that case, *Nfil3* deletion together with constitutive activation of CREB might help to improve regenerative axon growth. Secondly, the cellular and extracellular environment *in vivo* is very different from the *in vitro* situation. There may be many factors that interact with regenerating axons and change the gene expression response in injured DRG neurons such that the gene network state changes and the beneficial effects of *Nfil3* deletion cannot be expressed.

Taken together our data show that removal of the transcriptional repressor NFIL3 *in vivo* does not recapitulate the regeneration promoting effects that were previously observed *in vitro*. We know that NFIL3 acts together with other transcription factors, including CREB and C/EBPs, in a gene regulatory network that controls the expression of several regeneration-associated genes [12]. Apparently removal of NFIL3 *in vitro* is sufficient to alter the state of this network, whereas *in vivo* network control is more complex and more robust against external manipulation. On a more general note our data also indicate that *in vitro* data on the transcriptional regulation of axon growth may be difficult to translate to therapeutic intervention strategies for promoting neuronal regeneration *in vivo*.

## Supporting Information

S1 Table List of 5489 genes that are significantly regulated due to sciatic nerve lesion at either 2 or 5 days post-lesion. (XLSX)
